# In Silico Identification of Potential Quadruplex Forming Sequences in LncRNAs of Cervical Cancer

**DOI:** 10.3390/ijms241612658

**Published:** 2023-08-10

**Authors:** Deepshikha Singh, Nakshi Desai, Viraj Shah, Bhaskar Datta

**Affiliations:** 1 Department of Biological Engineering, Indian Institute of Technology Gandhinagar, Gandhinagar 382355, India; deepshikha.s@iitgn.ac.in (D.S.); nakshi.desai@alumni.iitgn.ac.in (N.D.); viraj.s.imsc14@ahduni.edu.in (V.S.); 2 Department of Chemistry, Indian Institute of Technology Gandhinagar, Gandhinagar 382355, India

**Keywords:** lncRNAs, G-quadruplex, putative quadruplex sequence, cervical cancer, G4-specific proteins

## Abstract

Long non-coding RNAs (lncRNAs) have emerged as auxiliary regulators of gene expression influencing tumor microenvironment, metastasis and radio-resistance in cancer. The presence of lncRNA in extracellular fluids makes them promising diagnostic markers. LncRNAs deploy higher-order structures to facilitate a complex range of functions. Among such structures, G-quadruplexes (G4s) can be detected or targeted by small molecular probes to drive theranostic applications. The in vitro identification of G4 formation in lncRNAs can be a tedious and expensive proposition. Bioinformatics-driven strategies can provide comprehensive and economic alternatives in conjunction with suitable experimental validation. We propose a pipeline to identify G4-forming sequences, protein partners and biological functions associated with dysregulated lncRNAs in cervical cancer. We identified 17 lncRNA clusters which possess transcripts that can fold into a G4 structure. We confirmed in vitro G4 formation in the four biologically active isoforms of SNHG20, MEG3, CRNDE and LINP1 by Circular Dichroism spectroscopy and Thioflavin-T-assisted fluorescence spectroscopy and reverse-transcriptase stop assay. Gene expression data demonstrated that these four lncRNAs can be potential prognostic biomarkers of cervical cancer. Two approaches were employed for identifying G4 specific protein partners for these lncRNAs and FMR2 was a potential interacting partner for all four clusters. We report a detailed investigation of G4 formation in lncRNAs that are dysregulated in cervical cancer. LncRNAs MEG3, CRNDE, LINP1 and SNHG20 are shown to influence cervical cancer progression and we report G4 specific protein partners for these lncRNAs. The protein partners and G4s predicted in lncRNAs can be exploited for theranostic objectives.

## 1. Introduction

Non-coding RNA (ncRNA), as the name suggests, are the RNAs that do not code for any protein. These sequences outnumber protein-coding sequences in the human genome. These nucleic acids were once considered dark matter and rendered unimportant due to their perceived disconnect from the central dogma [1]. However, non-coding RNAs have now assumed prominence for their gene-regulation roles. The largest group of ncRNAs includes transcripts that are over 200 nucleotides long and are termed long non-coding RNAs (lncRNAs). The spatiotemporal expression of lncRNAs across cell types has been correlated with several key cellular functions such as replication, transcription, translation, immune response, angiogenesis and apoptosis [2]. Further, the dysregulated expression of lncRNA transcripts has been correlated with various pathological conditions including cancer [3]. Cancer is a complex disease that alters the genomic and proteomic homeostasis of the cell to promote growth and proliferation [4]. The identification of specific biomarkers has revolutionized the early detection of cancers. Many cancers are curable if diagnosed at an early stage followed by suitable and timely treatment [5]. Nevertheless, cervical cancer causes the second highest number of deaths among women in India [6]. This is an alarming statistic considering that most cervical cancers can be successfully treated and human papillomavirus (HPV)-induced cancer can be prevented by vaccines [7]. The role of lncRNAs in tumorigenesis, metastasis and radio-resistance has compelled researchers to study them as potential cancer biomarkers [8]. While the precise mechanism by which lncRNAs control cancer dynamics is largely unknown, regulatory lncRNAs can serve as biomarkers of malignancies in different cancer phenotypes [9]. LncRNAs are notable for their heterogeneity, with sequence conservation across species ranging from very high to none. Moreover, sequence conservation does not guarantee functional resemblance in lncRNAs. Therefore, it is more intuitive to postulate a structure–function relationship for lncRNAs which allows them to access multiple binding sites for proteins, miRNA, mRNA, etc. [10]. LncRNAs range in length from a few hundred to several thousand nucleotides, folding into a plethora of complex secondary structures including G4s. rG4 structures are stabilized by K^+^ ions. The abundance of K^+^ ions inside human cells likely facilitates the adoption of G4 structures by RNA in relation to other RNA secondary structures [11,12]. Nevertheless, G4s have been suggested to maintain a dynamic equilibrium in vitro, between unfolded and folded states. Such equilibria appear likely in vivo with favorable intracellular K^+^ concentrations and the presence of helicases capable of resolving the structures. The G4RP-seq technique developed by Yang et al. supports the existence of transient G4-RNA in the human transcriptome. The study reported that lncRNAs avoid G4 formation under normal conditions in the absence of G4-binding ligands and when a lncRNA such as Metastasis Associated Lung Adenocarcinoma Transcript 1 (MALAT1) folds spontaneously into G4, then it is immediately countered or resolved by helicases and RNA-binding proteins (RBPs) [13]. Many reports have emerged that suggest implications of G-quadruplexes in key cancer-linked lncRNAs [9,12]. G-Quadruplex Forming Sequence Containing LncRNA (GSEC) was one of the first lncRNAs identified bearing a G-quadruplex structure and its importance in GSEC-mediated colorectal cancer cell migration was elucidated [14]. LINC00273, LncRNA In Non-Homologous End Joining Pathway 1 (LINP1), Nuclear Paraspeckle Assembly Transcript 1 (NEAT1), and Lung Cancer-Associated Transcript 1 (LUCAT1) are examples of other lncRNAs which are proposed biomarkers in different types of cancer and that execute their function via G4 secondary structures [15,16,17,18].

The in silico identification of G4s in lncRNAs is challenging because RNA folding/structure-prediction algorithms do not explicitly account for putative G4 sequences. The Vienna RNA folding suite estimates RNA G4 folding energy and assesses the competition between G4 folded and alternative RNA secondary structures [19]. However, there is a limitation to sequence input in such predictive tools, and the fact that lncRNAs are up to several thousand nucleotides long cannot be accepted as query sequences. In this work, we present a workflow that enables in silico identification of potential quadruplex-forming sequences in lncRNAs of cervical cancer. Subsequent in vitro analysis validates the G4-forming potential of our present in silico lncRNA predictions. As part of our in silico pipeline, we present two approaches to predict protein-interacting partners of cognate lncRNAs. We have strategically deployed several tools and databases with the goal of recognizing G4-forming lncRNAs in cervical cancer with potential prognostic capabilities. The overall workflow in the present work is illustrated in the graphical abstract. The G4-predicting algorithm QGRS rates the ability of dysregulated lncRNAs that have been initially identified on their potential to form G4s. The subsequent clustering of lncRNAs consolidates transcript variants of each lncRNAs for the rest of this study. The functionally relevant lncRNAs within each cluster are identified using BLAST. The G4-forming capability of the lncRNAs that have been thus shortlisted is assessed and validated by a combination of CD spectroscopy, ThT fluorescence and RT stop assays. Two different in silico approaches are deployed on the G4-bearing lncRNAs to identify protein-interacting partners and shed light on their potential regulatory functions.

## 2. Results

### 2.1. Identification of G-Quadruplex-Harboring Dysregulated LncRNAs in Cervical Cancer

We identified a total of 785 lncRNA transcript sequences as being mis-regulated in cervical cancer. After multi-sequence alignment, 622 unique lncRNA transcript sequences were shortlisted as input for QGRS mapper analysis. We obtained 47 transcript sequences after validation of G4-forming potential using non-B database. These were then segregated into lncRNA clusters. We obtained 14 lncRNA clusters at the end of our in silico screening methodology. The distribution of data and filtering of lncRNA can be seen in Supplementary Figure S1. Table 1 presents a list of lncRNA clusters identified in cervical cancer after meta-analysis. Notably, one lncRNA cluster can have more than one lncRNA transcript sequence depending on the splicing of its introns. For the remainder of this article, all mentions of the lncRNAs Maternally Expressed Gene 3 (MEG3), LncRNA In Non-Homologous End Joining Pathway 1 (LINP1), Small Nucleolar RNA Host Gene 20 (SNHG20) and Colorectal Neoplasia Differentially Expressed (CRNDE) refer to their functionally active isoforms. Furthermore, these physiologically relevant lncRNA isoforms were again subjected to QGRS analysis while maintaining the same query parameters. The lncRNAs that harbor G4-forming sequences with a G-score over 60 are listed in Supplementary Table S2. We used the corresponding isoform sequences to synthesize G4-possessing RNA transcripts for in vitro experiments.

### 2.2. In Vitro Characterization of PQS in Identified LncRNA Clusters

The previously identified 14 lncRNA clusters possess 45 PQS tracts. While we intend to comprehensively scrutinize all these eventually, in the present study, we have restricted our examination to four lncRNA clusters, MEG3, CRNDE, LINP1 and SNHG20. These four lncRNA clusters have G-scores ranging from 69 to 71 and possess different PQS-containing transcripts and expression patterns, thereby representing a varied sample set. The selected RNA sequences are listed in Supplementary Chart S1. We used the cognate PQS-RNA oligonucleotides for analyzing their potential to fold into stable G4 structures under cellular mimicking conditions. While MEG3, LINP1 and SNHG20 lncRNAs have 1 PQS, CRNDE has 2 PQS with G-score > 60. MEG3 is the only downregulated lncRNA among these four lncRNA clusters. Table 2 shows the RNA oligonucleotide sequences used in our study.

**Figure 1:**
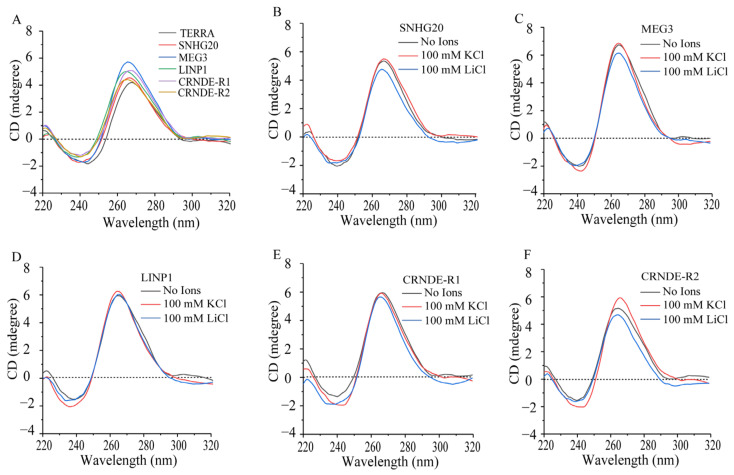
(**A**) Comparative CD spectra from PQS of lncRNA oligonucleotides (5 µM). CD spectra of lncRNAs (**A**) TERRA, (**B**) SNHG20, (**C**) MEG3, (**D**) LINP1, (**E**) CRNDE-R1, (**F**) CRNDE-R2, in the presence and absence of monovalent cations K^+^ (100 mM) and Li^+^ (100 mM)**.** Samples were prepared in G4-folding buffer as described in Section 4.

CD spectra of the lncRNA sequences (Figure 1A) suggest that all the chosen RNA molecules adopt parallel G4 with characteristic CD maxima at 265 nm and minima at 240 nm. We measured the CD spectra of the selected lncRNAs in the presence of monovalent cations such as K^+^ and Li^+^. Figure 1A–F depict the CD spectra of the lncRNAs in the presence and absence of cations K^+^ and Li^+^. While parallel topology of G4s is evident in the presence of ions, these are not found to exert a pronounced effect on change in G4 topology.

**Figure 2:**
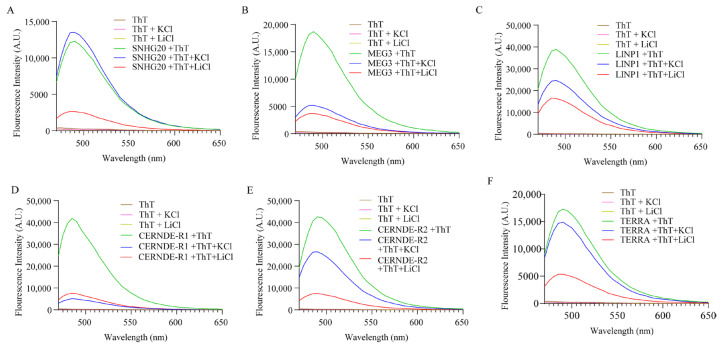
Emission spectra of (**A**) SNHG20, (**B**) MEG3, (**C**) LINP1, (**D**) CRNDE-R1, (**E**) CRNDE-R2 and (**F**) TERRA in the presence and absence of K^+^ and Li^+^ and ThT (2 µM) when excited at 445 nm.

**Figure 3:**
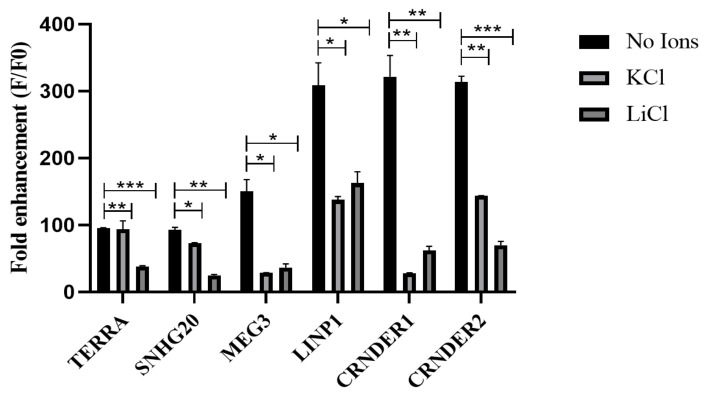
Fold enhancement of ThT fluorescence for lncRNAs in the presence and absence of monovalent cations, with excitation and emission at 445 and 488, respectively. Data expressed as mean ± SEM. * *p* ≤ 0.05; ** *p* ≤ 0.001, *** *p* ≤ 0.0001.

**Figure 4:**
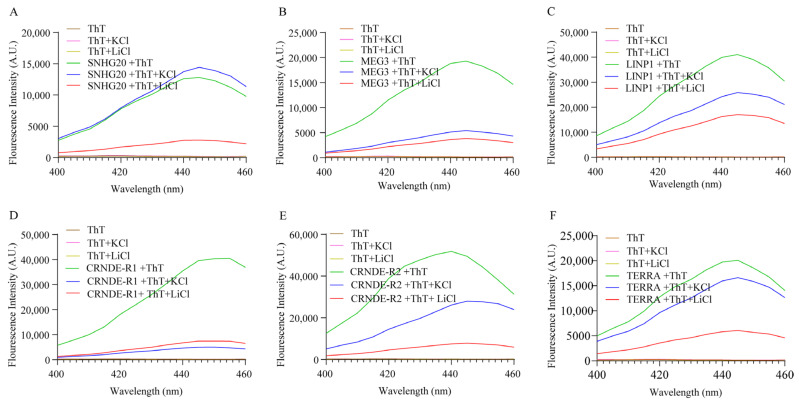
Excitation spectra of (**A**) SNHG20, (**B**) MEG3, (**C**) LINP1, (**D**) CRNDE-R1, (**E**) CRNDE-R2 and (**F**) TERRA in the presence and absence of K^+^ and Li^+^ and ThT (2 µM) with emission captured at 488 nm.

We next investigated the ability of these RNA G4 structures to respond to Thioflavin T (ThT). Figure 2 shows the emission spectra of ThT in the presence of various RNA sequences. ThT exhibits negligible fluorescence emission at 488 nm when dissolved in a buffer containing 1 M Tris and 0.5 M EDTA. An emission of a maximum of 490 nm was observed in all cases. ThT fluorescence was enhanced by about 300-fold in the presence of the G4 structures present in the lncRNAs LINP1, CRNDE R1 and CRNDE R2 and ~90 and ~150-fold, respectively, in the presence of SNHG20 and MEG3 (see Figure 3). These results clearly point to the formation of stable RNA G4s under the experimental conditions. While the presence of Li^+^ lowers the ThT emission across all the RNA sequences studied, the presence of K^+^ negatively affects the ThT emission in the case of MEG3, LINP1 and CRNDE-R1. The results of ThT emission assay for SNHG20 are closest to the expected behavior of G4s, with respect to superior enhancement in presence of K^+^ versus Li^+^ as observed in the positive control TERRA. To a modest extent, the results of ThT emission obtained with CRNDE-R2 also follow the expected behavior of greater G4 stabilization in the presence of K^+^ as opposed to Li^+^. The ThT excitation spectra with various RNA G4s display a similar pattern of fluorescence in comparing the presence versus absence of K^+^ and Li^+^ (Figure 4). These results preclude excited state artifacts of ThT in the ThT emission experiments described above and support the possibility of alternate G4 topologies in MEG3 and CRNDE-R1 that do not facilitate ThT binding.

### 2.3. RNA G-Quadruplex Structures Are Stabilized by the Presence of Monovalent Cations

**Figure 5:**
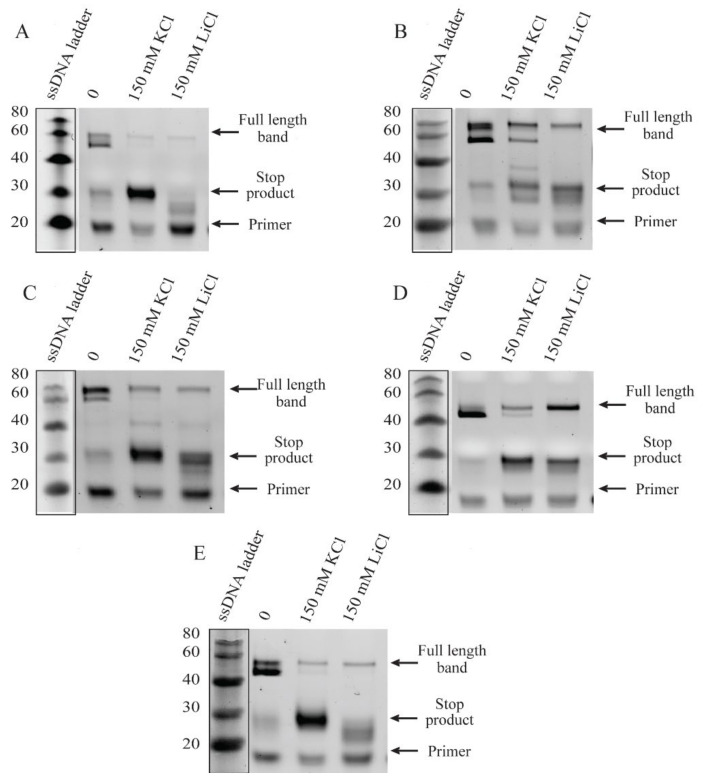
Reverse-transcriptase stop assay of (**A**) SNHG20, (**B**) MEG3, (**C**) LINP1, (**D**) CRNDE-R1 and (**E**) CRNDE-R2 in the presence of 150 mM of KCl and LiCl.

While the ThT fluorescence assay on RNA G4s depict an interesting and unexpected effect of monovalent ions, the indirect character of the assay could result in misleading inferences. We performed the reverse-transcriptase (RT) stop assay on the selected RNA sequences to develop another perspective on the role of monovalent ions. The RT stop assay was performed in the presence of monovalent cations, i.e., KCl and LiCl (150 mM). As shown in Figure 5, the RT stop assay performed on the selected lncRNAs displays two full-length products. Inspection of the denaturing page gel obtained in the absence of monovalent ions indicates that the intensity of the full-length product is higher than the stop product. In contrast, in the presence of K^+^ ions, the full-length product intensity decreased and stop product intensity increased. The greater amount of stop product in the presence of K^+^ suggests a stabilizing effect of the same on the G4 structure of the RNA. Notably, a similar pattern was observed in the presence of Li^+^ ions. However, the decrease in the full-length product intensity in the presence of Li^+^ was not as prominent as in the case of K^+^ ions. This suggests that the net stabilizing role of Li^+^ is lower compared to K^+^. These results reaffirm the outcomes of CD and ThT fluorescence experiments discussed previously. Nevertheless, the RT stop experiments are more sensitive in detecting the stabilizing effect of K^+^ on the RNA G4s. Our experiments successfully validate the in silico G4 identification in dysregulated lncRNAs of cervical cancer.

### 2.4. Protein-Interacting Partners and Co-Expression Network of Selected LncRNAs

Based on the dysregulated lncRNAs selected as per our in silico workplan, we decided to computationally predict the corresponding protein-interacting partners. We used two approaches to predict the protein-interacting partners of these lncRNAs. For reference, the FASTA sequence information for all proteins mentioned in the manuscript is provided in Supplementary Chart S2.

In our first, top-to-bottom approach, we begin with information from a database called lnc2catlas. Table 3 lists the cervical cancer-associated protein-interacting partners of the four selected lncRNAs with their corresponding interaction scores. PTEN, SMAD4, TP53 and CDKN2A are the four proteins identified to bind with corresponding lncRNAs LINP1, MEG3, CRNDE and SNHG20. SNHG20 is seen to interact with two proteins, TP53 and CDKN2A. SNHG20 and TP53 display the highest interaction score of 339.1, while CRNDE and TP53 show the lowest interaction score of 106.82. This analysis suggests that TP53 binds with all four lncRNAs.

**Figure 6:**
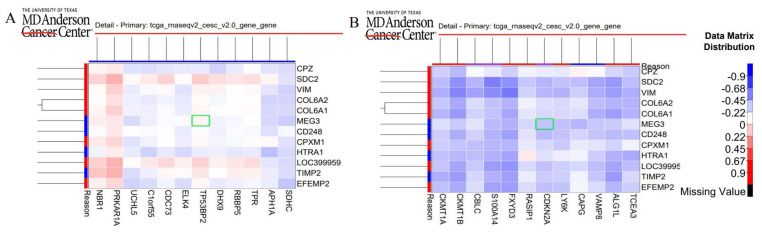
Heatmap for covariation between (**A**) MEG3 and TP53 and (**B**) MEG3 and CDKN2A (Highlighted in green box).

We performed a co-variation analysis to investigate the connectivity of lncRNA expression and TP53/CDKN2A expression. We selected an RNA expression platform for CESC cancer type and analyzed a gene probe/gene probe heatmap for identifying covariation between lncRNA and proteins (TP53 and CDKN2A). In the heatmap shown in Figure 6, we have placed protein transcripts on the x-axis and selected lncRNAs on the y-axis with correlation ranging from −1 to +1. Values closer to zero indicate an absence of a linear correlation between the two variables. Similarly, the values closer to +1 and −1 indicate positive and negative correlations between variables, respectively. Scrutiny of the heat map reveals that while MEG3 expression may not have a linear correlation with TP53 expression, it is negatively correlated with CDKN2A expression. The present heatmap was unable to provide information on other lncRNAs under study, possibly due to database update issues. Furthermore, protein–lncRNA interaction prediction is more confident when the subcellular localization is the same for lncRNA and protein transcript. Therefore, we evaluated the subcellular location of the lncRNAs and proteins under study. 

### 2.5. Protein-Interacting Partners for LncRNAs Using Bottom-to-Top Approach

We next performed a bottom-to-top approach based on literature reports for the identification of RNA G4-interacting proteins and computational prediction of their interaction with the lncRNAs under study. Table 4 shows the RPIseq scores of lncRNA with a corresponding binding protein, with a higher score indicating a greater likelihood of participation of a strong binding partner. The output score of >0.5 suggests a significant probability of interaction between lncRNA and the respective protein. Furthermore, the subcellular location of lncRNA and RBP has to be convergent to facilitate the binding. Based on these criteria, we have filtered out lncRNA-binding proteins shown in Table 4.

### 2.6. Identifying LncRNA and RNA-Binding Protein Localization

**Figure 7:**
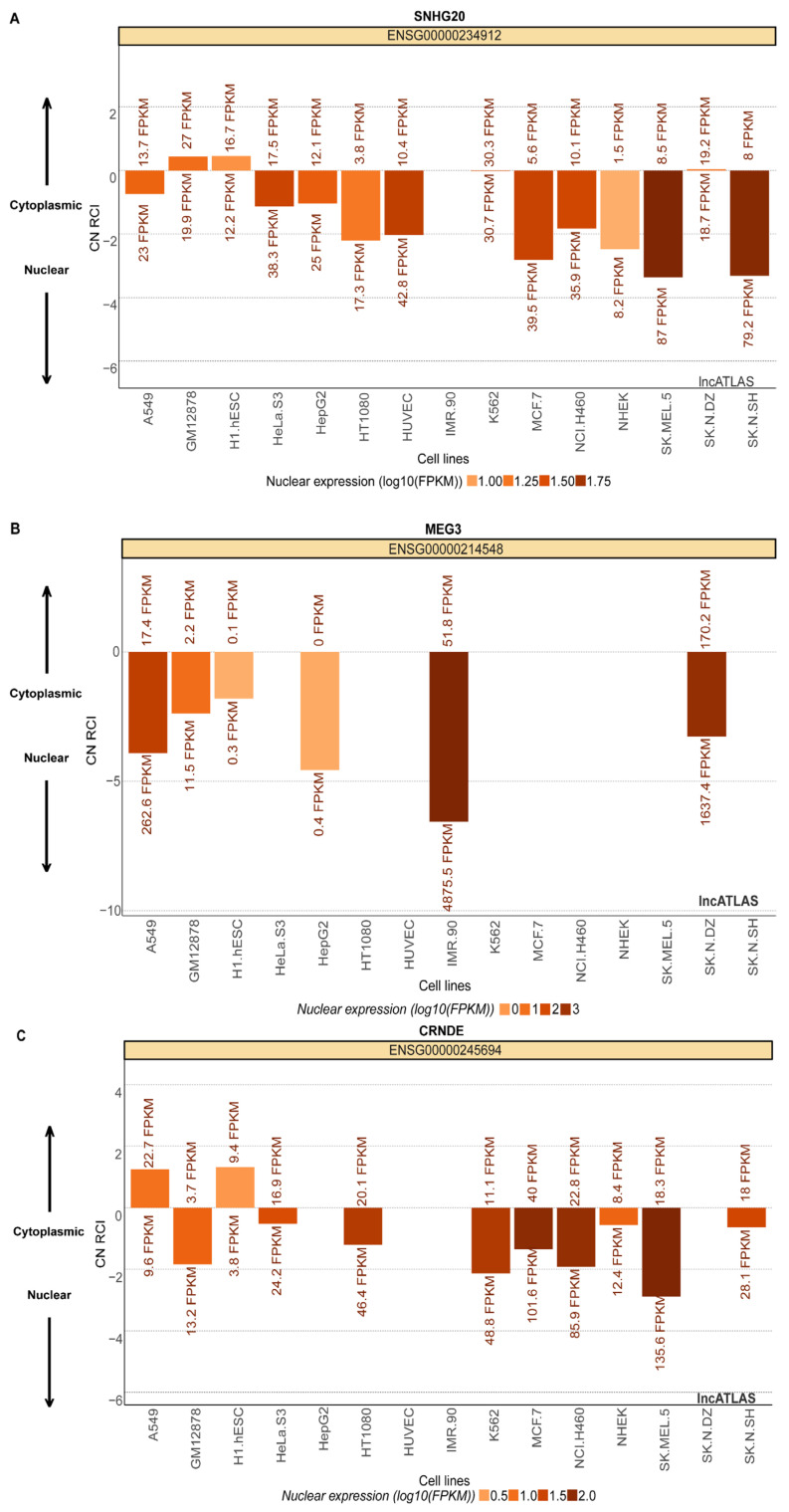
Subcellular localization of lncRNAs as obtained from lncATLAS database for (**A**) SNHG20, (**B**) MEG3 and (**C**) CRNDE.

We identified subcellular localization of lncRNAs using the lncATLAS database (Figure 7). Based on our investigation, we identified that MEG3, CRNDE and SNHG20 are localized in the nucleus (Figure 7A–C), while LINP1 is localized in the cytoplasm and perinuclear space. We extracted information related to subcellular localization of RBPs from the PROTEIN ATLAS database. Table 5 lists lncRNAs that have the highest probability of interacting with cognate RBPs identified by the bottom-to-top approach along with the biological function of those RBPs. The localization of the proteins shortlisted in the top-to-bottom approach are as follows: PTEN and SMA4 are localized in nucleoplasm and cytosol, TP53 is localized in nucleoplasm and CDKN2A is localized in nucleoli. Thus, all four proteins bear a high probability of physical interaction with lncRNAs. We have also listed downstream biological functions of the lncRNAs under study in cervical cancer as obtained from the lnc2cancer database (Table 6). 

## 3. Discussion

This work is based on two primary objectives: (1) in silico identification of PQSs present in dysregulated lncRNAs of cervical cancer, and (2) in silico enunciation of G4-specific RNA-binding proteins that are likely to associate with the RNAs obtained from objective (1). The first part of our work highlights the feasibility of deploying appropriate in silico prediction methodologies for identifying G-quadruplex-forming sequences in hitherto-unexplored nucleic acid contexts. Exploration of G4 structures originated from experimental information about the behavior of specific motifs that could also be considered as reference points. The advent of multiple data repositories and structure-prediction algorithms has made it possible to develop ab initio reference points first before prioritizing experimental follow-up. At the end of our in silico pipeline, we identified 14 lncRNA clusters (Table 1). A few lncRNAs in this list, notably MALAT1 and NEAT1, have been studied for their regulatory roles in cancer progression [28,29,30]. An interesting aspect of our approach is the treatment of transcript variants of the lncRNAs selected for further scrutiny and experimental validation. It is known that there are 12 alternatively spliced variants of CRNDE, of which CRNDE-g is a highly expressed isoform in multiple cancer types [31]. SNHG20, on the other hand, has only one variant which is upregulated in cancer and possesses a G4-forming site [32]. Similarly, LINP1 has one predominantly expressed isoform known to adopt a stable G4 structure [33]. In contrast, MEG3 is downregulated in cancer and has many physiologically expressed isoforms, while we are studying the variant that has PQS [34]. Thus, the G4s being considered in the selected lncRNAs are part of functional isoforms and make our findings substantive.

We experimentally validated in silico predictions by a combination of CD spectroscopy, ThT fluorescence assay and reverse-transcriptase (RT) stop assay. As demonstrated by the in vitro experiments, the predicted putative quadruplex sequences in the four selected lncRNAs form stable G4s. Different RNA G4 topologies exhibit distinctive CD signals [35,36]. The orientation of strands and the molecularity of the G4s are major influences on the geometry of G4s, based on the hydrogen-bonding requirements of G-quartets and the chemical-bonding constraints of the nucleosides. CD is sensitive to the geometry of G4s and is commonly used to classify them as parallel, anti-parallel or mixed [37]. The chosen RNA molecules adopt parallel G-quadruplexes according to their respective CD spectra. The variations in CD intensities can be attributed to varied sequence lengths, subtleties in loop lengths and overall architecture resulting in some variation in the stabilities of corresponding quadruplexes [38,39]. It is well known that monovalent cations can stabilize G4 structures by coordinating the O6 atom in the G-quartet channel. The inability of cations such as Li^+^ to stabilize G4 formation, in contrast to the supportive role of physiologically relevant Na^+^ and K^+^ cations, is widely used to scrutinize the G4-forming behavior of oligonucleotides [40]. Notably, our results suggest that while Li^+^ impairs the G4-folding ability of all the selected RNAs, the presence of K^+^ is most beneficial for the G4 formed by SNHG20. 

**Figure 8:**
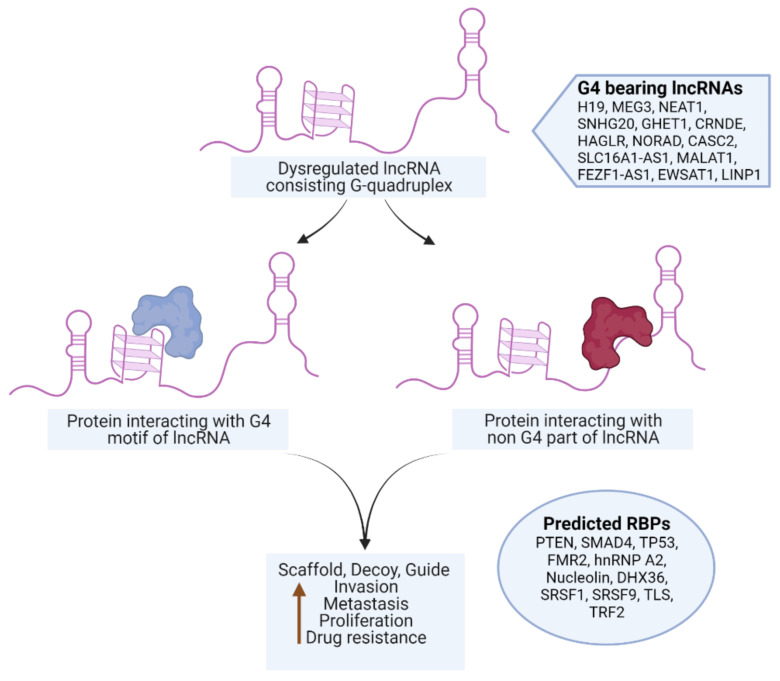
Identification of G4-bearing lncRNAs and prospective protein partners of G4-bearing lncRNAs that are dysregulated in cervical cancer.

Interestingly, the fluorescence enhancement of ThT was weakened in the presence of the monovalent ions for specific RNAs. While the effect of monovalent cations on DNA G4s is widely deployed as a canonical assessment of quadruplex stability, similar interpretations of RNA G4 behavior are not straight-forward. The architecture of G-tracts and spacer lengths in the MEG3, LINP1 and CRNDE-R1 sequences being tested suggest potential for polymorphism in the corresponding G4s in the presence of specific monovalent ions [41,42]. Considering that the CD spectra of these sequences are not significantly perturbed in the presence versus absence of K^+^ or Li^+^, it is possible that the parallel G4s being formed arise from a different number of participating RNA molecules. Moreover, the ThT assay relies on the dye’s ability to bind in end-stacking mode, and G4 topologies that do not provide easy access for end-stacking may be mis-identified as unstable G4s [43,44]. The results of ThT fluorescence assay and the RT stop assay on the selected RNAs indicates the subtle similarity in the behavior of G4s of SNHG20 and CRNDE-R2 on the one hand and MEG3, LINP1 and CRNDE-R1 on the other. The presence of two template bands in the RT-stop assay is attributable to 5′ and 3′ heterogeneity in the RNA obtained by in vitro transcription [45,46]. While the primary objective of our in vitro experiments was to validate the in silico searching approach, our results also point to the subtleties in in vitro behavior of the RNA G4s based on the sequence characteristics of the corresponding RNA PQSs. The value of identification and validation of G4-bearing lncRNAs in the first part of our work can be better appreciated from Figure 8. The G4 motifs in the lncRNAs that emerge from our in silico pipeline, and that are validated through in vitro experiments, project lncRNAs, such as SNHG20, that have hitherto not been studied in the context of their secondary structure and protein interaction via such constructs in cervical cancer. 

As part of our second objective, we tested two approaches to predict G4-specific RBPs that are likely to interact with the lncRNAs under study. LncRNAs are purported to exert distinctive effects via interaction with partners such as proteins, DNA, mRNA or even other lncRNAs [47]. Among these, the identification of protein-interacting partners of a dysregulated lncRNA is likely to be of value in dissecting molecular pathways underlying cancer progression. LncRNAs have been shown to act as guides, signals, decoys and scaffolds for many proteins [48]. RBPs are critical for regulatory RNAs to exert their cellular functions. Nevertheless, lncRNA–protein interaction can be orchestrated in many ways other than binding such as via allosteric regulatory molecules and miRNAs [49]. Proteins such as PRC1/2, WDR5, SMAD2/3 and HnRNP are known to interact with different lncRNAs. Such lncRNA–protein associations can be connected to disease inception and propagation, thereby also providing diagnostic and therapeutic strategies for the corresponding diseases [50,51,52,53].

We employed both top-to-bottom and bottom-to-top approaches. In the top-to-bottom approach, we utilized a database called lnc2catlas, which resulted in four RBPs, TP53, CDKN2A, PTEN and SMAD4, that are ranked and categorized based on a score and their association with specific cancer types. Heatmaps were employed to analyze the co-occurrence patterns between lncRNAs and proteins. Literature mining has revealed that LINP1 does not bear the TP53-binding site to directly regulate its cellular function but p53 regulates the expression and function of LINP1 [54]. We could not find reports confirming the direct interaction or binding of SNHG20 and CRNDE with TP53 or CDKN2A. MEG3 can interact with the p53 DNA-binding domain and its intact structure is important for p53-mediated transactivation [55]. The negative correlation of MEG3 with CDKN2A is consistent with literature reports that suggest that the downregulation of MEG3 and overexpression of CDKN2A in cervical cancer is involved in disease progression [56].

The inability of the top-to-bottom approach to focus exclusively on G4-binding proteins led us to test a converse bottom-to-top approach to identify the proteins that interact with RNA G4 structures. This approach relied on previously reported RNA G4-binding proteins, including FMR2, hnRNP A2, Nucleolin, DHX36, SRSF1, SRSF9, TLS and TRF2. It is intuitive to assume that the probability of binding between a lncRNA and a protein would be higher if they shared the same subcellular location. Therefore, we examined the subcellular locations of the selected lncRNAs and their interacting proteins. The in silico predictions showed colocalization between RNA-protein pairs that had attractive scores in the RPISeq analysis. Consequently, these proteins have a significant likelihood of physically interacting with lncRNAs. LINP1 is the only lncRNA having cytoplasmic presence and is known to translocate to the nucleus in response to DNA damage [33]. It may also serve as a possible interacting partner for FMR2 and DHX36. It is worthwhile to consider that FMR2 has a nuclear localization signal and can be translocated into the nucleus or nuclear speckles if triggered by regulatory molecules [20]. Therefore, FMR2 can also be a plausible interacting partner for CRNDE, MEG3 and SNHG20. The main takeaway from these results is the selective proteins postulated to interact with lncRNAs which can be further evaluated by in vivo proteomics experiments. The interaction of these proteins with specific lncRNAs may trigger activation or inhibition of downstream pathways that will ultimately contribute to tumor progression. The selected lncRNAs primarily participate in cell growth, epithelial-to-mesenchymal transition and apoptosis (Table 5). Notably, among the listed RBPs in Table 5, DHX36 has been previously reported to actively resolve G4s [57,58]. The other RBPs that were identified in our search are yet to be reported in direct contact with RNA G4s. Thus, these results could be used as motivation for conducting detailed experimental analyses of RBP–protein interactions.

The value of the results obtained in the second part of our work can also be better appreciated from Figure 8. The G4 motifs in the lncRNAs that emerge from our in silico pipeline and that are validated through in vitro experiments may or may not be directly involved in associating with proteins. The presence of G4 motifs in these lncRNAs essentially serves as a “hook” to identify a host of proteins that partner with the lncRNA, and would otherwise have remained inaccessible due to the severe constraints of systematic experimental assessment. Such information is valuable for understanding the possible roles played by specific lncRNA. For example, SNHG20 is one of the four lncRNAs that we have examined for its ability to possess G4 folding sites. The identification of SNHG20 led to the subsequent prediction of interactions with TP53 and CDKN2A. Targeted experiments that probe SNHG20 interaction with TP53, CDKN2A or other proteins are likely to shed light on the biological role of SNHG20 in cervical cancer progression, which is currently not understood.

The in silico predictions in this work do not replace experimental validation. Instead, they support the in silico approach and provide a framework for systematic experimental investigations. In future, experimental validation of protein-interacting partners identified by the approach reported in this work would facilitate further scrutiny of their diagnostic and therapeutic potentials. Notably, alterations in quadruplex structure using synthetic ligands can potentially disrupt or stabilize the tertiary structure of lncRNAs, thereby affecting the lncRNA–protein partner interactions and providing a therapeutic handle. Our laboratory is currently pursuing these G4-mediated activities of dysregulated lncRNAs in cervical cancer.

## 4. Materials and Methods

### 4.1. Bioinformatic Prediction of Putative G4-Forming Sequences, G4-Protein Interactions and Localization

#### 4.1.1. Selection of LncRNAs Dysregulated in Cervical Cancer

Lnc2cancer (http://bio-bigdata.hrbmu.edu.cn/lnc2cancer/ assessed on 1 June 2020) is a manually curated database which has a list of lncRNAs experimentally supported as bearing association with specific cancers [59]. The nucleotide sequences used in our study were obtained from the first version of the Lnc2cancer database and were subjected to ExPASy analysis for validating their non-coding nature. All lncRNAs were subjected to multi-sequence alignment using clustalW for transcript sequences obtained from Lnc2cancer, Ensembl and NCBI. To identify predominant lncRNA isoforms, we performed nucleotide BLAST with the help of primer sequences of lncRNAs derived from literature reports. Next, we filtered out the non-identical lncRNA transcript sequences because of low confidence in the corresponding sequence architecture. Lnc2cancer has since been updated to version 3.0, containing embedded links for Refseq and Ensembl FASTA sequences [60,61]. Each lncRNA can display several transcript variants as a result of alternative splicing. In the present work, we define every lncRNA comprising all its variants as one lncRNA cluster.

#### 4.1.2. Prediction of PQS

QGRS mapper (https://bioinformatics.ramapo.edu/QGRS/analyze.php, assessed on 10 December 2022) was used for the prediction of PQS in our work. QGRS mapper is an established algorithm that identifies putative G-quadruplex-forming sequences (PQS) in input nucleotide sequences [62]. This tool factors in important features such as the maximum G4 length and loop size, assigning scores to each potential sequence to rank them and determine the most probable sequence when multiple alternatives exist. We adopted the following parameters for QGRS mapper analysis: maximum length: 45, minimum G-group: 3 and loop length: 1–14. Non-overlapping sequences with a G-scope of over 60 were chosen for further processing. High-scoring sequences are understood to be better candidates for G4 folding. We validated the G4-forming potential of nucleotide sequences filtered as above, using Non-B Database (https://nonb-abcc.ncifcrf.gov/apps/site/default assessed on 10 December 2022). This database contains comprehensive information on all mammalian genomic regions that are predicted to adopt alternate structures to B-DNA such as Z-DNA, quadruplex-forming motifs, mirror repeats, inverted repeats and direct repeats with subsets of cruciform, triplex and slipped structures [63].

#### 4.1.3. Prediction of LncRNA-Protein Interaction

Lnc2Catlas (http://lnc2catlas.bioinfotech.org/, assessed on 10 June 2020) provides interactions between 33 different cancers and 27,670 lncRNA transcripts [64]. This database sorts interacting protein partners of lncRNA based on a score and classifies them according to cancer type. We also performed covariation analysis between lncRNA and protein-interacting partners using Heatmap for data across all cancer types using the TCGA Next-Generation Clustered Heat Map (NG-CHM) in CESC (Cervical Squamous Cell Carcinoma) in RNA expression platform for gene vs. gene heatmap. We analyzed heatmaps from TCGA Next-Generation Clustered Heat Map (NG-CHM) Compendium available at https://bioinformatics.mdanderson.org/TCGA/NGCHMPortal/, assessed on 10 January 2023. This compendium includes 297 interactive Next-Generation Clustered Heat Maps (NG-CHMs) for exploring cancer bioinformatics data from The Cancer Genome Atlas (TCGA) project. Our choice of proteins as part of our bottom-to-top approach was based on 8 RNA G4-binding proteins reported by Brazda et al. These are FRAXE-associated Mental Retardation Protein (FMR2), Heterogeneous Nuclear Ribonucleoproteins A2 (hnRNPA2), Nucleolin, DEAH Box Protein 36 (DHX36), Serine/Arginine-Rich Splicing Factor 1 (SRSF1), Serine/Arginine-Rich Splicing Factor 9 (SRSF9), protein Translocated in Liposarcoma (TLS) and Telomeric Repeat Binding factor 2 (TRF2) [65].

We also used the database RPIseq for predicting RNA–protein interactions using only sequence information. The RPIseq server (http://pridb.gdcb.iastate.edu/RPISeq/, assessed on 15 January 2023) can predict the probability that a specific protein and RNA interact and is based on a family of machine learning classifiers.

#### 4.1.4. Prediction of LncRNA and Interacting Protein Localization

PROTEIN ATLAS (https://www.proteinatlas.org/, assessed on 20 January 2023) was used to obtain the localization of the proteins that interact with lncRNAs. To detect the localization of lncRNAs, LncATLAS was used. LncATLAS (https://lncatlas.crg.eu/, assessed on 20 January 2023) is an easy-to-use web-based visualization tool for obtaining useful information about expression localization of lncRNAs [66].

### 4.2. Oligonucleotides and Compounds

The oligonucleotide sequences used for the experimental studies are listed in Table 2 and were synthesized using an in vitro transcription method using a T7 promoter based on a modification of the conventional protocol [67]. The T7 promoter sequence is slightly modified in such a way that there is good yield, low 5′ heterogeneity and the Gs in the promoter sequence do not interfere with the G4 sequence. The oligonucleotide sequences used for in vitro transcription are mentioned in Supplementary Table S1. The sense DNA strand of T7 RNA promoter and antisense DNA strand of T7 RNA promoter with oligo were annealed together as per the protocol provided by Sigma-Aldrich, St. Louis, MO, USA. The concentration and purity of annealed DNA oligonucleotides were quantified using NanoDrop™ 2000 spectrophotometer (Thermo Fisher Scientific, Waltham, MA, USA). In vitro transcription of the annealed DNA oligonucleotides was carried out using HiScribe™ T7 High Yield RNA Synthesis Kit (New England Biolabs, Ipswich, MA, USA), following the manufacturer’s protocol with a slight modification. Dithiothreitol is added to the reaction mixture to stabilize enzymes. The DNA oligonucleotides in the transcribed RNA solution were digested using DNase I, RNase-free (Thermo Fisher Scientific, USA). RNAs were cleaned and eluted using Monarch^®^ RNA Cleanup Kit (500 μg) (New England Biolabs, USA), following the manufacturer’s protocol. The concentration and purity of eluted RNAs were quantified using a NanoDrop™ 2000 spectrophotometer (Thermo Fisher Scientific, USA) and were stored at −80 °C until further use.

### 4.3. CD Spectroscopy

Circular Dichroism (CD) spectroscopy was used to evaluate potential G4 formation in RNA sequences. CD spectra were recorded on a JASCO J-815 spectrophotometer and all measurements were carried out at 16 °C in the wavelength range of 220–350 nm, using a response time of 1 s, a step size of 1 nm and a 2 nm bandwidth. The scanning speed of the instrument was set at 100 nm/min, with an average of three scans. A 10 mm path length quartz cuvette was used in all experiments. Samples containing 5 μM RNA were folded in a buffer containing 10 mM Tris-Cl (pH 7.5) and 10 mM Tris-Cl (pH 7.5), 0.01 mM EDTA (pH 8.0) by incubating at 95 °C for 5 min and cooled to room temperature before CD analysis.

### 4.4. Fluorescence Spectroscopy

ThT has been suggested as an efficient reporter for distinguishing between G4 and non-G4 RNA structures [68]. Fluorescence enhancement assays were performed using Thioflavin T (ThT) (Sigma-Aldrich, USA) as an RNA G4-binding dye in a 96-well black fluorescence microplate. RNA samples (2 µM) were folded in the presence or absence of ions in a buffer containing 10 mM Tris-Cl (pH 7.5) and 10 mM Tris-Cl (pH 7.5), 0.01 mM EDTA (pH 8.0) by incubating at 95 °C for 5 min followed by gradually cooling to room temperature over 2 h. ThT (2 μM) was added to the folded RNA G4 and excitation spectra were obtained with emission captures at 488 nm, while the emission spectra were obtained after excitation at 445 nm. Single-point fluorescence intensities were also obtained for ThT at the mentioned wavelengths. The fluorescence of samples was measured at 25 °C using Cytation 5 Cell Imaging Multimode Reader (Agilent Technologies, Santa Clara, CA, USA).

### 4.5. Reverse-Transcriptase Stop Assay

As per the design of the RT stop assay, the RNA template is translated by the reverse transcriptase enzyme, up until it encounters a stable RNA G4 structure. Truncated complement DNA products are created and can be visualized by denaturing PAGE assay [44]. Texas Red-tagged primers were purchased from Sigma Aldrich, USA in lyophilized form and nuclease-free water was used to prepare 100 µM solutions. Each RT-stop experiment was performed in 10 μL reaction mixtures containing 2 μM RNA, 100 nM Texas Red-tagged primer, 2 mM NTPs and KCl/LiCl (150 mM). The tagged primer and RNAs were annealed by first denaturing by heating at 95 °C for 5 min, then cooling to room temperature over 2 h. Reverse transcriptase was added to the reaction and incubated for 1 h at 37 °C. The reverse-transcriptase reaction was stopped using a buffer consisting of 95% Formamide, 0.05% Bromophenol Blue, 20 mM EDTA, and 0.05% Xylene cyanol. The products were separated on a 15% denaturing (UREA) polyacrylamide gel, visualized on a ChemiDocTM MP Imaging system using the Rhodamine filter and then counter-stained with DiamondTM Nucleic Acid dye (Promega Corporation, San Luis Obispo, CA, USA) to visualize template bands. 

### 4.6. Statistical Analysis

For statistical analysis, an unpaired *t*-test was carried out. Statistical significance is shown with asterisks: * *p* ≤ 0.05; ** *p* ≤ 0.001, *** *p* ≤ 0.0001.

## 5. Conclusions

In this study, we have outlined an in silico method to predict and analyze cervical cancer specific G-quadruplex-bearing lncRNAs. From among 14 different lncRNAs that are considered to possess G4 motifs, we have experimentally characterized the G4 formation by 4 lncRNAs, namely, SNHG20, MEG3, CRNDE and LINP1. As part of this study, we have profiled the RNA-binding proteins that are likely to interact with these lncRNAs, playing important roles in the progression of cervical cancer. Based on the outcome of this work, we suggest G4 motifs as an attractive structural element that could be used to identify dysregulated lncRNAs in cervical cancer and their interacting proteins as potential biomarkers. While this study does not purport to sacrifice experimentation, it offers a workable plan for identifying and prioritizing dysregulated lncRNA-based experiments that seek to shed light on their mechanistic or functional links in cervical cancer progression. Identification of lncRNA–protein axes using the approach presented here could be valuable from diagnostic and therapeutic perspectives, and researchers in relevant domains are thus likely to find value in the in silico approach.

## Figures and Tables

**Table 1:** LncRNA clusters obtained after in silico meta-analysis of the Lnc2cancer database.

Sr. No.	LncRNA Cluster Name	PQS Containing Isoforms for Each LncRNA Cluster	Expression	Max G-Score in PQS
1	H19	7	upregulated	144
2	MEG3	5	downregulated	70
3	NEAT1	6	upregulated	72
4	SNHG20	1	upregulated	72
5	GHET1	1	upregulated	64
6	CRNDE	1	upregulated	71
7	HAGLR	6	upregulated	71
8	NORAD	5	upregulated	71
9	CASC2	1	downregulated	65
10	SLC16A1-AS1	1	differential	61
11	MALAT1	4	upregulated	70
10	FEZF1-AS1	1	upregulated	63
13	EWSAT1	4	downregulated	69
14	LINP1	1	upregulated	69

**Table 2:** RNA oligonucleotides used for performing in vitro studies.

Name	Sequence (5′ to 3′)	G-Score	Length
TERRA	GGGTTAGGGTTAGGGTTAGGG	72	60
SNHG20	GGGTTTGGGCTGGGGCCTGGG	72	36
MEG3	GGGAAATTCTCAGGAGGGGGACCTGGGCCAAGGG	64	40
LINP1	GGGGTAGGAGAGGGTATGGGGACCAGGGCACTCTGTAAGGG	69	30
CRNDE R1	GGGCTAGGGCCTGGGCCTCGGG	71	34
CRNDE R2	GGGTGTCGGGGTTCGGGGCGGG	72	33

**Table 3:** Protein-interacting partners from Lnc2catlas for the lncRNAs under study.

LncRNA	Protein	Protein Transcript	Score	Cancer Type
LINP1	PTEN	PTEN-001	213.74	CSCC and EA *
LINP1	PTEN	PTEN-001	213.74	CSCC and EA
LINP1	TP53	TP53-001	165.3	CSCC and EA
MEG3	SMAD4	SMAD4-001	308.18	CSCC and EA
MEG3	TP53	TP53-001	249.83	CSCC and EA
CRNDE	TP53	TP53-001	106.82	CSCC and EA
CRNDE	TP53	TP53-001	106.82	CSCC and EA
SNHG20	TP53	TP53-001	339.1	CSCC and EA
SNHG20	CDKN2A	CDKN2A-001	273.52	CSCC and EA
SNHG20	TP53	TP53-001	339.1	CSCC and EA

* Table abbreviations: CSCC: Cervical Squamous Cell Carcinoma; EA: Endocervical Adenocarcinoma.

**Table 4:** A summary of postulated binding probability between lncRNA and cognate RNA-binding proteins.

LncRNA	Binding Protein	RF-Score ^a^	SVM-Score
LINP1	FMR2	0.8	0.91
	hnRNP A2	0.85	0.72
	Nucleolin	0.9	0.946
	DHX36	0.8	0.989
	SRSF1	0.95	0.947
	SRSF9	0.8	0.922
	TLS	0.9	0.869
	TRF2	0.85	0.948
CRNDE	FMR2	0.75	0.99
	hnRNP A2	0.9	0.85
	Nucleolin	0.95	0.981
	DHX36	0.75	0.997
	SRSF1	0.95	0.978
	SRSF9	0.75	0.968
	TLS	0.9	0.945
	TRF2	0.8	0.983
SNHG20	FMR2	0.8	0.81
	Nucleolin	0.8	0.702
	DHX36	0.75	0.961
	SRSF1	0.8	0.657
	SRSF9	0.7	0.71
	TRF2	0.85	0.68
MEG3	FMR2	0.8	0.91
	hnRNP A2	0.7	0.9
	Nucleolin	0.85	0.574
	DHX36	0.7	0.824
	SRSF1	0.8	0.511

^a^ Predictions with probabilities >0.5 can be considered “positive”, indicating the corresponding RNA and protein are likely to interact.

**Table 5:** Summary of subcellular localization of RNA-binding proteins derived from the PROTEIN ATLAS database and cognate biological functions from the literature.

RNA-Binding Protein	LncRNAs	Subcellular Localization	Biological Function
FMR2	LINP1	Cytoplasm	Transcriptional regulation [20]
hnRNP A2	MEG3, CRNDE, LINP1	Nucleoplasm/Nucleus	Maturation, transport and metabolism of mRNA [21]
Nucleolin	MEG3, SNHG20, CRNDE, LINP1	Nucleoplasm/Nucleus	Facilitates chromatin transcription, chromatin remodeling [22]
DHX36	LINP1, MEG3, SNHG20, CRNDE	Nucleoplasm and Cytoplasm	Helicase resolving RNA G-quadruplexes [23]
SRSF1	MEG3, SNHG20, CRNDE, LINP1	Nucleoplasm/Nucleus	Regulating mRNA transcription, stability and nuclear export, translation and protein sumoylation [24]
SRSF9	SNHG20, CRNDE, LINP1	Nucleoplasm/Nucleus	mRNA processing, mRNA splicing [25]
TLS	CRNDE, LINP1	Nucleoplasm/Nucleus	DNA repair, transcription, protein translation, RNA splicing and transport [26]
TRF2	SNHG20, CRNDE, LINP1	Nucleoplasm/Nucleus	DNA binding, double-stranded telomeric DNA binding, protein homodimerization activity, telomeric DNA binding, telomerase activity [27]

**Table 6:** Summary of lncRNA functions in cervical cancer from lnc2cancer database.

LncRNA	Biological Function
MEG3	Cell growth, epithelial-to-mesenchymal transition
LINP1	Cell growth, apoptosis
CRNDE	Cell growth, epithelial-to-mesenchymal transition, apoptosis
SNHG20	Not known

## Data Availability

Most data generated or analyzed during this study are included in this published article and its Supplementary Materials files. Other datasets used and/or analyzed during the current study are available from the corresponding author on reasonable request.

## Supplementary Materials

The supporting information can be downloaded at: https://www.mdpi.com/article/10.3390/ijms241612658/s1.
