# Supplementary material for: In Silico Identification of Potential Quadruplex Forming Sequences in LncRNAs of Cervical Cancer
Source: Int J Mol Sci. 2023 Aug 10;24(16):12658. doi: 10.3390/ijms241612658 (PMC10454738; doi:10.3390/ijms241612658)
Supplement: Supplementary file 1 [file ijms-24-12658-s001.zip › ijms-2501831-supplementary.pdf]

Electronic Supplementary Information

# *In silico* identification of potential quadruplex forming sequences in lncRNAs of cervical cancer

Deepshikha Singh[1, ‡], Nakshi Desai[1, ‡], Viraj Shah[1] and Bhaskar Datta[*,1,2]

[1]Department of Biological Engineering, [2]Department of Chemistry, Indian Institute of Technology Gandhinagar, Gandhinagar, Gujarat, India

* **Corresponding author**

* Bhaskar Datta, Department of Biological Engineering, Indian Institute of Technology Gandhinagar, Gandhinagar 382355 India.

‡ These authors contributed equally to the work.

Telephone: 079-2395-2427; Fax: 079-2397-2622; E-mail: bdatta@iitgn.ac.in

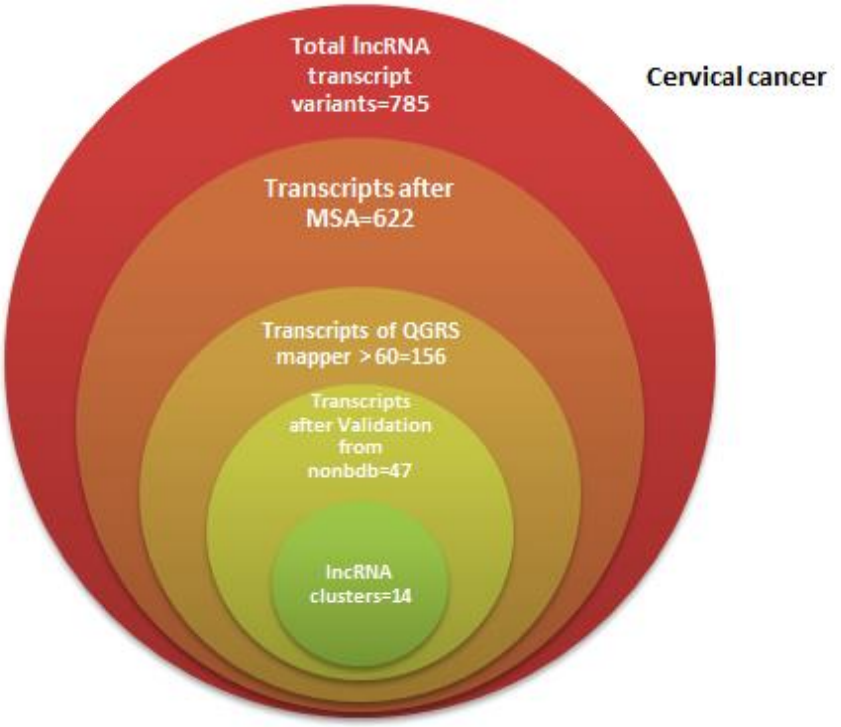


**Supplementary Figure S1:** Distribution of lncRNAs for cervical cancer at different stages of *in silico* analysis.

**Supplementary Table S1:** DNA oligonucleotides used for in vitro transcription

| lncRNA name | Sequence |
|---|---|
| TERRA | TGTGTTCTTGTTCTCTGTCGTTGTTTTGTGCCCTAACCCTAACCCTAACCCTAATTTCGCTATAGTGAGTCGTATTA |
| SNGH20 | TGTGTTCTTGTTCTCTGTCGTTGTTTTGTGCCCAGGCCCCAGCCCAAACCCTTTCGCTATAGTGAGTCGTATTA |
| MEG3 | TGTGTTCTTGTTCTCTGTCGTTGTTTTGTGCCCTTGGCCCAGGTCCCCCTCCTGAGAATTTCCCTTTCGCTATAGTGAGTCGTATTA |
| LINP1 | TGTGTTCTTGTTCTCTGTCGTTGTTTTGTGCCCTTACAGAGTGCCCTGGTCCCCATACCCTCTCCTACCCCTTTCGCTATAGTGAGTCGTATTA |
| CRNDE-R1 | TGTGTTCTTGTTCTCTGTCGTTGTTTTGTGCCCGAGGCCCAGGCCCTAGCCCTTTCGCTATAGTGAGTCGTATTA |
| CRMDE-R1 | TGTGTTCTTGTTCTCTGTCGTTGTTTTGTGCCCGCCCCGAACCCCGACACCCTTTCGCTATAGTGAGTCGTATTA |
| T7 promotor sequence | TGTGTTCTTGTTCTCTGTCGTTGTTTTGTG |

**Supplementary Table S2:** LncRNA isoforms in clusters and the QGRS analysis of the transcript sequence. QGRS analysis parameters used were as follows- Max Length: 45; Min G group: 3; Loop size: 1-14.

| **lncRNA H19** | NCBI ID | PQS found | Position | Length | PQS | G-score |
|---|---|---|---|---|---|---|
| Variant 1 | NR_002196.2 | 2 | 14 | 26 | **GGGGGGTGGGGGGTGGGGGTGGGGGG** | 144 |
| | | | 879 | 36 | **GGGCAGTGCAGGGTGTCACCAGAAGGGGAGTGGGGG** | 67 |
| Variant 2 | NR_131223 | 2 | 14 | 26 | **GGGGGGTGGGGGGTGGGGGTGGGGGG** | 144 |
| | | | 879 | 36 | **GGGCAGTGCAGGGTGTCACCAGAAGGGGAGTGGGGG** | 67 |
| | | 2 | 14 | 26 | **GGGGGGTGGGGGGTGGGGGTGGGGGG** | 144 |
| | | | 879 | 36 | **GGGCAGTGCAGGGTGTCACCAGAAGGGGAGTGGGGG** | 67 |

| **lncRNA MEG3** | NCBI ID | PQS found | Position | Length | PQS | G-score |
|---|---|---|---|---|---|---|
| Variant 1 | NR_002766.2 | 1 | 561 | 34 | **GGGAAATTCTCAGGAGGGGGACCTGGGCCAAGGG** | 64 |
| Variant 2 | NR_003530.2 | 1 | 561 | 34 | **GGGAAATTCTCAGGAGGGGGACCTGGGCCAAGGG** | 64 |
| Variant 3 | NR_003531.3 | 1 | 537 | 34 | **GGGAAATTCTCAGGAGGGGGACCTGGGCCAAGGG** | 64 |
| Variant 4 | NR_033358.1 | 1 | 561 | 34 | **GGGAAATTCTCAGGAGGGGGACCTGGGCCAAGGG** | 64 |
| Variant 5 | NR_033359.1 | 1 | 561 | 34 | **GGGAAATTCTCAGGAGGGGGACCTGGGCCAAGGG** | 64 |
| Variant 6 | NR_033360.1 | 1 | 561 | 34 | **GGGAAATTCTCAGGAGGGGGACCTGGGCCAAGGG** | 64 |
| Variant 7 | NR_046464.1 | 1 | 561 | 34 | **GGGAAATTCTCAGGAGGGGGACCTGGGCCAAGGG** | 64 |
| Variant 8 | NR_046465.2 | 1 | 561 | 34 | **GGGAAATTCTCAGGAGGGGGACCTGGGCCAAGGG** | 64 |
| Variant 9 | NR_046466.1 | 1 | 561 | 34 | **GGGAAATTCTCAGGAGGGGGACCTGGGCCAAGGG** | 64 |
| Variant 10 | NR_046467.1 | 1 | 561 | 34 | **GGGAAATTCTCAGGAGGGGGACCTGGGCCAAGGG** | 64 |
| Variant 12 | NR_046469.1 | 1 | 561 | 34 | **GGGAAATTCTCAGGAGGGGGACCTGGGCCAAGGG** | 64 |
| Variant 13 | NR_046470.2 | 1 | 561 | 34 | **GGGAAATTCTCAGGAGGGGGACCTGGGCCAAGGG** | 64 |

| | | | | | | |
|---|---|---|---|---|---|---|
| Variant 14 | NR_046471.1 | 1 | 561 | 34 | **GGGAAATTCTCAGGAGGGGGACCTGGGCCAAGGG** | 64 |
| Variant 15 | NR_046472.1 | 1 | 561 | 34 | **GGGAAATTCTCAGGAGGGGGACCTGGGCCAAGGG** | 64 |
| Variant 16 | NR_046473.1 | 9 | 561 | 34 | **GGGAAATTCTCAGGAGGGGGACCTGGGCCAAGGG** | 64 |
| | | | 3077 | 37 | **GGGTCCTCCTGATGAGGGTGTTGGGGTCCCCCCTGGG** | 65 |
| | | | 3693 | 31 | **GGGAGGGAGGAGTGGGGCCACAGAGAGAGGG** | 62 |
| | | | 3878 | 38 | **GGGCATTGTAGACAGGGATGAGGGTCATTGATCCTGGG** | 65 |
| | | | 6706 | 37 | **GGGGGCAGCAGAGGGTCAGGGAGCAATAGGCCCTGGG** | 62 |
| | | | 7453 | 33 | **GGGAGCACGTGGGAGGCAGGGGAGTGGGGTGGG** | 70 |
| | | | 7539 | 21 | **GGGTGGGATGGGGAGTGCGGG** | 68 |
| | | | 7810 | 43 | **GGGCCACATGAGCTGGGCCTCGTGGGCCTGATGTGGTGCTGGG** | 64 |
| | | | 8001 | 20 | **GGGGCGGGAGGGGTTCAGGG** | 70 |

| **lncRNA NEAT 1** | NCBI ID | PQS found | Position | Length | PQS | G-score |
|---|---|---|---|---|---|---|
| NEAT1 Variant Menbeta | NR_131012.1 | 11 | 14 | 33 | **GGGAGGGATGCGCGCCTGGGTGTAGTTGTGGGG** | 63 |
| | | | 425 | 30 | **GGGTGCAGGGCTGCAGGAGGGCCGGGAGGG** | 68 |
| | | | 2070 | 28 | **GGGAGTTGTGGGGGACGGGGAGAAGGGG** | 71 |
| | | | 2171 | 27 | **GGGATGAGGGTGAAGAAGGGGAGAGGG** | 69 |
| | | | 2250 | 38 | **GGGAATTCTCTGGGGCTTTGGGGAATTTAGTGCGTGGG** | 66 |
| | | | 3226 | 34 | **GGGAAGGGGATGGGGATTGTGGGGAGAGAGTGGG** | 71 |
| | | | 4559 | 34 | **GGGGAGATAAGGGCTGAACTGGGACTAGCCAGGG** | 71 |
| | | | 19560 | 33 | **GGGGTGGGGACCTTTGGGCTCTGACCCGAAGGG** | 63 |
| | | | 21342 | 35 | **GGGATGATGGGGCTTGAGCAAAGTGGGGGAGGGGG** | 65 |
| | | | 21866 | 40 | **GGGTGGCCTGGGGCAAGTACCGGCTGAGGGTCCAGGTGGG** | 65 |

| | | | | | | |
|---|---|---|---|---|---|---|
| | | | 22619 | 15 | **GGGAGGGAGGGAGGG** | 72 |
| NEAT1 Variant MENEPSILON | NR_028272.1 | 6 | 14 | 32 | **GGGAGGGATGCGCGCCTGGGTGTAGTTGTGGG** | 63 |
| | | | 425 | 30 | **GGGTGCAGGGCTGCAGGAGGGCCGGGAGGG** | 68 |
| | | | 2070 | 28 | **GGGAGTTGTGGGGGACGGGGAGAAGGGG** | 71 |
| | | | 2171 | 27 | **GGGATGAGGGTGAAGAAGGGGAGAGGG** | 69 |
| | | | 2250 | 38 | **GGGAATTCTCTGGGGCTTTGGGGAATTTAGTGCGTGGG** | 66 |
| | | | 3226 | 34 | **GGGAAGGGGATGGGGATTGTGGGGAGAGAGTGGG** | 71 |

| **lncRNA SNHG20** | NCBI ID | PQS found | Position | Length | PQS | G-score |
|---|---|---|---|---|---|---|
| Transcript 1 | NR_027058.1 | 1 | 1143 | 21 | **GGGTTTGGGCTGGGGCCTGGG** | 72 |

| **lncRNA GHET1** | NCBI ID | PQS found | Position | Length | PQS | G-score |
|---|---|---|---|---|---|---|
| Transcript 1 | NR_130107.1 | 1 | 1171 | 43 | **GGGAAGGGGGGTTGGTGCCATTCTGGGAGAGGAGGTTTCTGGG** | 64 |

| **lncRNA CRNDE** | NCBI ID | PQS found | Position | Length | PQS | G-score |
|---|---|---|---|---|---|---|
| Variant 1 | NR_034105.4 | 0 | - | - | - | - |
| Variant 2 | NR_034106.3 | 2 | 230 | 22 | **GGGCTAGGGCCTGGGCCTCGGG** | 71 |
| | | | 279 | 22 | **GGGTGTCGGGGTTCGGGGCGGG** | 70 |
| Variant 3 | NR_110453.2 | 0 | - | - | **-** | - |
| Variant 4 | NR_110454.2 | 2 | 230 | 22 | **GGGCTAGGGCCTGGGCCTCGGG** | 71 |
| | | | 279 | 22 | **GGGTGTCGGGGTTCGGGGCGGG** | 70 |
| Variant 5 | NR_170995.1 | 2 | 230 | 22 | **GGGCTAGGGCCTGGGCCTCGGG** | 71 |
| | | | 279 | 22 | **GGGTGTCGGGGTTCGGGGCGGG** | 70 |

| **lncRNA HAGLR** | NCBI ID | PQS found | Position | Length | PQS | G-score |
|---|---|---|---|---|---|---|
| | | | | | | |
| Variant 1 | NR_110458.1 | 1 | 927 | 39 | **GGGCCAGTGGGCTGGTACAGACTAGGGAAGAGAAAAGGG** | 64 |

| | | | | | | |
|---|---|---|---|---|---|---|
| Variant 2 | NR_110459.1 | 2 | 102 | 31 | **GGGCTGCCTGGGCAAGGGCGGGGCGAGAGGG** | 71 |
| | | | 878 | 39 | **GGGCCAGTGGGCTGGTACAGACTAGGGAAGAGAAAAGGG** | 64 |
| Variant 3 | NR_110460.1 | 2 | 102 | 31 | **GGGCTGCCTGGGCAAGGGCGGGGCGAGAGGG** | 71 |
| | | | 878 | 39 | **GGGCCAGTGGGCTGGTACAGACTAGGGAAGAGAAAAGGG** | 64 |
| Variant 4 | NR_110461.1 | 1 | 783 | 39 | **GGGCCAGTGGGCTGGTACAGACTAGGGAAGAGAAAAGGG** | 64 |

| **lncRNA NORAD** | NCBI ID | PQS found | Position | Length | PQS | G-score |
|---|---|---|---|---|---|---|
| Variant 1 | NR_027451.1 | 5 | 79 | 30 | GGGCCGGCGGGTGAACTGGGGGGCCCCGGG | 70 |
| | | | 346 | 27 | GGGCGGCGCTGGGCGTGGTGGGCCGGG | 67 |
| | | | 3482 | 28 | GGGTTGGTAGAGGTGGGAAAGGGAAGGG | 63 |
| | | | 4098 | 23 | GGGGTGGGGGAAGGGGGGGTGGG | 71 |
| | | | 4377 | 42 | GGGATGGTGGAAGAGGGAGAAGAGGAAGGGTGAAGGGAAGGG | 70 |

| **lncRNA CASC2** | NCBI ID | PQS found | Position | Length | PQS | G-score |
|---|---|---|---|---|---|---|
| Variant 1 | NR_026939.1 | 2 | 114 | 34 | **GGGAACGTGAAGGGGCGGGGCCTCGAGGTCAGGG** | 64 |
| | | | 193 | 40 | **GGGCGCAATGGCGGGGACCCGGGGCTTGATGCTGCTTGGG** | 65 |
| Variant 2 | NR_026940.1 | 2 | 114 | 34 | **GGGAACGTGAAGGGGCGGGGCCTCGAGGTCAGGG** | 64 |
| | | | 193 | 40 | **GGGCGCAATGGCGGGGACCCGGGGCTTGATGCTGCTTGGG** | 65 |
| Variant 3 | NR_026941.1 | 2 | 114 | 34 | **GGGAACGTGAAGGGGCGGGGCCTCGAGGTCAGGG** | 64 |
| | | | 193 | 40 | **GGGCGCAATGGCGGGGACCCGGGGCTTGATGCTGCTTGGG** | 65 |

| **lncRNA SLC16A1-AS1** | NCBI ID | PQS found | Position | Length | PQS | G-score |
|---|---|---|---|---|---|---|
| Transcript 1 | NR_103743.1 | 1 | 549 | 29 | **GGGTGGGGAGACTGGCCCAGTGGGTTGGG** | 61 |

| **lncRNA MALAT1** | NCBI ID | PQS found | Position | Length | PQS | G-score |
|---|---|---|---|---|---|---|
| Variant 1 | NR_002819.4 | 6 | 590 | 31 | **GGGCCGTGGGGGGCTGGCGGCAACTGGGGGG** | 60 |
| | | | 3790 | 35 | **GGGATGGTCTTAACAGGGAAGAGAGAGGGTGGGGG** | 63 |
| | | | 4851 | 27 | **GGGTGGGCTTTTGTTGATGAGGGAGGG** | 60 |
| | | | 6348 | 24 | **GGGAAGGGAGGGGGTGCCTGTGGG** | 67 |
| | | | 6535 | 22 | **GGGATGGGAGGAGGGGGTGGGG** | 70 |
| | | | 7726 | 24 | **GGGGAGGGAAAGGGGGAAAGCGGG** | 69 |
| Variant 2 | NR_144567.1 | 6 | 590 | 31 | **GGGCCGTGGGGGGCTGGCGGCAACTGGGGGG** | 60 |
| | | | 3556 | 35 | **GGGATGGTCTTAACAGGGAAGAGAGAGGGTGGGGG** | 63 |
| | | | 4617 | 27 | **GGGTGGGCTTTTGTTGATGAGGGAGGG** | 60 |
| | | | 6114 | 24 | **GGGAAGGGAGGGGGTGCCTGTGGG** | 67 |
| | | | 6301 | 22 | **GGGATGGGAGGAGGGGGTGGGG** | 70 |
| | | | 7492 | 24 | **GGGGAGGGAAAGGGGGAAAGCGGG** | 69 |
| Variant 3 | | 5 | 590 | 28 | **GGGCCGTGGGGGGCTGGCGGCAACTGGG** | 60 |
| | | | 3556 | 35 | **GGGATGGTCTTAACAGGGAAGAGAGAGGGTGGGGG** | 63 |
| | | | 5871 | 24 | **GGGAAGGGAGGGGGTGCCTGTGGG** | 67 |
| | | | 6058 | 21 | **GGGATGGGAGGAGGGGGTGGG** | 70 |
| | | | 7249 | 24 | **GGGGAGGGAAAGGGGGAAAGCGGG** | 69 |

| **lncRNA FEZF1-AS1** | NCBI ID | PQS found | Position | Length | PQS | G-score |
|---|---|---|---|---|---|---|
| Transcript 1 | NR_036484.1 | 1 | **442** | 34 | **GGGAGCCGAGGGCACCGCCGCGGGCGCCGCCGGG** | 63 |

| **lncRNA EWSAT1** | NCBI ID | PQS found | Position | Length | PQS | G-score |
|---|---|---|---|---|---|---|
| Transcript 1 | NR_026949.1 | 1 | 1557 | 22 | **GGGAGCGGGCTGGCGGGGTGGG** | 69 |

| **lncRNA LINP1** | NCBI ID | PQS found | Position | Length | PQS | G-score |
|---|---|---|---|---|---|---|
| Transcript 1 | NR_138480.1 | 1 | 614 | 41 | **GGGGTAGGAGAGGGTATGGGGACCAGGGCACTCTGTAAGGG** | 69 |

**Supplementary Chart S2:** Sequences of RNAs selected for PQS identification

**SNHG20**

AAGUUGCUGACGGAGCUACUUCCGCCCAGGGGAGUGGGAAGUAAGUGGAGACACGUGCU
UUGGCCUGUUGGAGGGGAAACCCGCUCUCGCCUCCUGGUGGUCGCCGACUCGCAGUCCGC
AGGAUGACUCAGGGCAGCCCUGACCACAGUUCCGCCGCCAUCGGCCCUAGCCUGCGGAAU
UGGGCUCGCCCCGGGACGAUAACAGAGCUCUGCCGGGGGCUGGAGGCACUGACCGGGUG
ACCAGAGACCCAGAGACCAGACCCCCUCCACGGCGCCCGGGAUUUCGGGGACGGCUUCUC
CCAUCGCAAGUUUCAACAGAAAAUGAGAAAUAUCCCCCGACGAUUGGCUGAAAACAUGC
AGCAACCACUAUUUUCUUCCUGCCCCUCGUUGAUGAGAGCAUUCGAAGUGACCUCAGCA
GGGCAUCCAGGUCAGUUUCUGGAAGACUUGUGUGUGUGAUGAAUGAAUAUCUGGUUUU
GUCUCUGCUGGGCCUGUGUGCCUGGAAAGGAAUUGUUUUGGCCUAGGAUCAUCCAGGUU
UGUUUGGUUUAGUUCUUGACCACAUAUUUUUUGAAUGGUGACUGCUUAAGACCCUGUUG
UGUAUGGCUAUAAAUAGAUACACGCCAAGGUGACCACAUACUCUGUGGUUCCUGUGCCC
GCCGGCCAUGUGUCUGAGUGUGUAGCCUCUCAUCAUCUAAAGGAACUUUGGCUGCAGAG
GGGAGGCCUGGUCCCAUGGGAAGUUUUGGGAGCGCAGCAGCAGGUGGGUUCAAACGACC
CAGCAAGUGCCUCCUUAGCACUUAGAGGCGGAGGCCACCACGUUGUCCACGUGGGGUUU
CUGAUCCCAGCUCCCCACCAGCCUGCUGGACCUCGGGCAGGUCCCUCCCUGUUUGUACCU
CCAUUACUUCUUCAGUAAGAUGGGGACACUGAAGAUGACCAUGCCUCCCACCAGAUUGG
UGCUUUUUUUGUUUUAAUAAGGGACAGAGUCUCACUAUGUUGCCUAUAGGCUGGUCUCA
AGCUCUUGGCCUCAGUUUUCCUGCCUCAGCCUUCAAAUAUGCUGGGGUGACAGGCAUGA
GCCACUGCACUGGCCCAGAUUGGUACAUUUGAGGGUUAAAUGAGCAAAUCCUAUAAAGC
ACUCAGGAGAGGGCUGUGGGGUUUGGGCUGGGGCCUGGGAGCACUUUAGACGUGGUAGC
UAUGUUGUUGUCACCUUUCUCCUCUUUCAAAGCAUUUUUUGUUUGUUUGUUUUUGUUU
UUGAGACAGAGUUUCGCUCUUGUGGCCCAGGCUGGAGUGCGGUGGUGUGACCUCGGCUC
ACUGCAUCCUCCGCCUCCCGGGUUCAAGUGAUGGGAUUACAGGUGCCUGCCACAACGCCC
AGCUAAUUGUUUGUAUUUUUAGUAGAGACGAGGUUUCACCAUGUUGGCCAGGCUGGUCU
CGAACUCCUGACCUCAGGUGAUCCACCUGCCUUGGCCUCCCAAAGUGCUGGGAUUACAGG
UAUGAGCUACCGCGCCCGGCACAAAGCAUUUUCAGCUGUGAAAUUCCCUGGGGUGCUGC
AGUAGAGGAAGGUUUGGGGUCUUGACGGGUGCUAUUUGCCACGGAAAGAUGCCUUCCUG
CUCCCAGGGCAGGAGAGCCGAGGUAAGACUUACUGUAGGCUGUCGUUUUUUUUGUUUGU
UUUUUGUCUUUGCGAUGGAGUCUCACUCUGUCGCCAGGCUGGAGUGCAGUGGCAUGAUC
UUGGCUCACUGCAGAACCUCCACCUCCCAGGUUCAAGCGAUUCUCCUGCCUCAGCCUCCC
AAGUAACUGGGAUUACAGGCACAUGCCCCCACAACCAGCUAAUUUUUUAUUUUUAGUAG
AGACAGGGUUUCACAUGUUGGCCAGGCUGGUCUUGAACUCCUGACCUCAGGUGAUCCGC
CCGCCUCGGCCUCCCAAAGUGCUGGGAUUACAGACAUGAGCCACUGCGCCCAGCCAGGCU
GUUGUUUUUUUACCUCCUUGUUUGCACAAUUUGGGCCACUCACAAGAGUGUAUACCCUG
UGAUAAACAGUUACCUACAUUCUCCUCUGCAUGCUUGUCUUUAGAGGAAGGAAAUGUAU
UAAUUGCCCAAAGUAAUAUAUUGUGUUAAGAUGUGAUAUAUACUGGGGAAAAAAAAAG
UGUAUAUUGACAUUUCUGGAAUAAACCACUUUGAUUCCCAAAAAAAAAAAAAAA

**LINP1**

CCCUCCGUCUCCUUGACUCUGGGUGGGCUGUGUGACUUUUCCUUGCUCAAUAGAAUGAG
AAGGAGGUGGCGCACCGGCUACAAGGCUAGACCGGGGGCUCGCAUAUCUCCACUUGCAG
CUGCCACUGCCAUUAGAAGAACUUGCUCAGCCAGCUCCUGGGCCCCAGGAAAAGGACAA
GUGUCACCCAGAACAGAGCCACCUCCAGCUGCACUCAGCUCCAGAAGCUGGCCCAGCCGG
UCCAGUACACCUUUUAAAUAAUGUCCUCUACGUGCCGGUGUGGAAGUAGCCCGGAUGCA
AUUGAAUGAACAACAGACGGUGCUUUCCAGGACGGCGCUGUGCUUUCCAGGAUGGUGCU
GUGCUUUCAUUCAUUUGGGUAGCUCCUCUGUGAGCCUCCCAGCGCCGACUGCAGAGCCCC

CACUCUCCAGCCUGCAAGACCCCGAAAUUCAAGCCACACAAAGAAAGGAGGAGGGGCCG
UUGGCAUUUACUGAACCUUAUAAAACUGUCAGCAAAACAGCCCUUAGGCUUGGACUCCC
UGCUAGCCGGGUUUUACGGUGCUGAAGUCAGCAUCUUGAUUCAGCUGCAUAAAUAAUCU
CCUGCAGUCCUGCAAGGCCUGGGGUAGGAGAGGGUAUGGGGACCAGGGCACUCUGUAAG
GGCUGGGAUAGGAACCCCAGGGAAUAAGACAGACCAACUGCGGGACUUCAGACUCCACU
GCAGCCGGGAUCGGGUUGUUGUUAAUUUCUUAAGCAAUUUCUAAAUUCUGUAUUGACUC
UCUCAUGCAUGUAACUGAUCCUUAGAUAUUGUCAGCCAAAUGACUAAAGGAUUUAGAAA
UAAAAAAAA

**MEG3**

AGCCCCUAGCGCAGACGGCGGAGAGCAGAGAGGGAGCGCGCCUUGGCUCGCUGGCCUUGG
CGGCGGCUCCUCAGGAGAGCUGGGGCGCCCACGAGAGGAUCCCUCACCCGGGUCUCUCCU
CAGGGAUGACAUCAUCCGUCCACCUCCUUGUCUUCAAGGACCACCUCCUCUCCAUGCUGA
GCUGCUGCCAAGGGGCCUGCUGCCCAUCUACACCUCACGAGGGCACUAGGAGCACGGUUU
CCUGGAUCCCACCAACAUACAAAGCAGCCACUCACUGACCCCCAGGACCAGGAUGGCAAA
GGAUGAAGAGGACCGGAACUGACCAGCCAGCUGUCCCUCUUACCUAAAGACUUAAACCA
AUGCCCUAGUGAGGGGGCAUUGGGCAUUAAGCCCUGACCUUUGCUAUGCUCAUACUUUG
ACUCUAUGAGUACUUUCCUAUAAGUCUUUGCUUGUGUUCACCUGCUAGCAAACUGGAGU
GUUUCCCUCCCCAAGGGGGUGUCAGUCUUUGUCGACUGACUCUGUCAUCACCCUUAUGA
UGUCCUGAAUGGAAGGAUCCCUUUGGGAAAUUCUCAGGAGGGGGACCUGGGCCAAGGGC
UUGGCCAGCAUCCUGCUGGCAACUCCAAGGCCCUGGGUGGGCUUCUGGAAUGAGCAUGC
UACUGAAUCACCAAAGGCACGCCCGACCUCUCUGAAGAUCUUCCUAUCCUUUUCUGGGG
GAAUGGGGUCGAUGAGAGCAACCUCCUAGGGUUGUUGUGAGAAUUAAAUGAGAUAAAA
GAGGCCUCAGGCAGGAUCUGGCAUAGAGGAGGUGAUCAGCAAAUGUUUGUUGAAAAGGU
UUGACAGGUCAGUCCCUUCCCACCCCUCUUGCUUGUCUUACUUGUCUUAUUUAUUCUCC
AACAGCACUCCAGGCAGCCCUUGUCCACGGGCUCUCCUUGCAUCAGCCAAGCUUCUUGAA
AGGCCUGUCUACACUUGCUGUCUUCCUUCCUCACCUCCAAUUUCCUCUUCAACCCACUGC
UUCCUGACUCGCUCUACUCCGUGGAAGCACGCUCACAAAGGCACGUGGGCCGUGGCCCGG
CUGGGUCGGCUGAAGAACUGCGGAUGGAAGCUGCGGAAGAGGCCCUGAUGGGGCCCACC
AUCCCGGACCCAAGUCUUCUUCCUGGCGGGCCUCUCGUCUCCUUCCUGGUUUGGGCGGAA
GCCAUCACCUGGAUGCCUACGUGGGAAGGGACCUCGAAUGUGGGACCCCAGCCCCUCUCC
AGCUCGAAAUCCCUCCACAGCCACGGGGACACCCUGCACCUAUUCCCACGGGACAGGCUG
GACCCAGAGACUCUGGACCCGGGGCCUCCCCUUGAGUAGAGACCCGCCCUCUGACUGAUG
GACGCCGCUGACCUGGGGUCAGACCCGUGGGCUGGACCCCUGCCCACCCCGCAGGAACCC
UGAGGCCUAGGGGAGCUGUUGAGCCUUCAGUGUCUGCAUGUGGGAAGUGGGCUCCUUCA
CCUACCUCACAGGGCUGUUGUGAGGGGCGCUGUGAUGCGGUUCCAAAGCACAGGGCUUG
GCGCACCCCACUGUGCUCUCAAUAAAUGUGUUUCCUGUCUUAACAAAAA

**CRNDE**

GCAGCCGCAGCCCCUGGCGCUAACGGUCGGUAACGGCCCGCGCGCCGCCCGCCGGGGG
CUCGCGCCAGCCACGAGGGAGCGUCCGCGGCCCGCGCGCCCGCGCGGCGGAGGAGAGGUG
AGCCCCCGCCCGGGCCAGGCCCUCUGGCCGCGCCGUCCGCCCCUCUAGUCGUGUCCCCUCG
UGGGCCGAACGGACGCGGCGGUGCCCCGCGCCCGACCAGACGUCCCGUGGGCUAGGGCCU
GGGCCUCGGGCCGCGUCGGCGCCGGUCGAGCCUCUCCGGGUGUCGGGGUUCGGGGCGGGC
GCGCGUGGGCGUGGCUCCUCUGUCCACGCCUGUUCCCUUCGUCGCCGCGGCUCUCGUCCG
GGACACGGCUUUCCGGAGUAGAGCCCUUGGAGGUGUUAAGUGUGAUGCUUCCAUAAUAC
AUUUGGAUGCUGUCAGCUAAGUUCACUUCUGAACUAAGGGGUUCCUCCAAAUGUUGGCU
GAAAUUCAUCCCAAGGCUGGUCUGCAAAGUCUGCAAUUCAUAAUGGAGCUACUGUACUG
GCUAUUGGAAGGAGGAGAUUCUGAAGAUAAGGAGGUAAAACCUGUUUAGAAAUUAAAA

AUGAGUUACGAUUUAAAGAAAAUUCAGAUGACUCAUUGUGAGUGCUAGUUCUCUUGUA
GGAUGCCACUGGAAAUGUUGAAAUGAAAAAUAUUCAGCCGUUGGUCUUUGAAAUUUCCU
GUGAUGUGUUUCAAUCUAGAUGCAAAGAACAUGGAAAAAUCAAAGUGCUCGAGUGGUU
UAAAUAUGUUUUGGGUAUUCCUGUUUAUAGACUAUAAUACUUUUCCAAUUAAAAUCCU
CAGUUGUCACGCAGAAGAAGGUUAAGCUGUAUUUGAUUGCCAGUUUUACUGAAAAUGCU
UAGUAUUUUACAGUAUCACCAAAUAUAUUUUGUUUAGCCAAGGUAUAGGAAAAAUAAA
AUAAAUUGUAUAGGUUGACUUUUUUCUAAAAUGUCUUUAUUGGAUUGAAUGAAUGUUU
AUACCUGAAAAAAAAAGGUUCAAAAAAA

**Supplementary Chart S2:** FASTA Sequences of proteins under study in this work.

**>TP53**

WVDSTPPPGTRVRAVAIYKQSQHMTEVVRRCPHHERCSDSD

**>CDKN2A**

KGGAAAAELGPGGGENMVRRFLVTLRIRRACGPPRVRVFVVHISWFTGEWAAPGAPAAVALVLM
LLRSQRLGQQPLPRRPGHDDGQRPSGGAAAAPRRGAQLRRPRHSHPTRARRCPGGLPGHAGGAA
PGRGAAGRARCLGPSARGPG

**>NUCLEOLIN**

MVKLAKAGKNQGDPKKMAPPPKEVEEDSEDEEMSEDEEDDSSGEEVVIPQKKGKKAAATSAKKV
VVSPTKKVAVATPAKKAAVTPGKKAAATPAKKTVTPAKAVTTPGKKGATPGKALVATPGKKGAA
IPAKGAKNGKNAKKEDSDEEEDDDSEEDEEDDEDEDEDEDEIEPAAMKAAAAAPASEDEDDEDD
EDDEDDDDDEEDDSEEEAMETTPAKGKKAAKVVPVKAKNVAEDEDEEEDDEDEDDDDDEDDED
DDDEDDEEEEEEEEEEEPVKEAPGKRKKEMAKQKAAPEAKKQKVEGTEPTTAFNLFVGNLNFNKSA
PELKTGISDVFAKNDLAVVDVRIGMTRKFGYVDFESAEDLEKALELTGLKVFGNEIKLEKPKGKDSK
KERDARTLLAKNLPYKVTQDELKEVFEDAAEIRLVSKDGKSKGIAYIEFKTEADAEKTFEEKQGTEID
GRSISLYYTGEKGQNQDYRGGKNSTWSGESKTLVLSNLSYSATEETLQEVFEKATFIKVPQNQNGKS
KGYAFIEFASFEDAKEALNSCNKREIEGRAIRLELQGPRGSPNARSQPSKTLFVKGLSEDTTEETLKES
FDGSVRARIVTDRETGSSKGFGFVDFNSEEDAKAAKEAMEDGEIDGNKVTLDWAKPKGEGGFGGR
GGGRGGFGGRGGGRGGRGGFGGRGRGGFGGRGGFRGGRGGGGDHKPQGKKTKFE

**>DHX36 isoform 1**

MSYDYHQNWGRDGGPRSSGGGYGGGPAGGHGGNRGSGGGGGGGGGGRGGRGRHPGHLKGREI
GMWYAKKQGQKNKEAERQERAVVHMDERREEQIVQLLNSVQAKNDKESEAQISWFAPEDHGYG
TEVSTKNTPCSENKLDIQEKKLINQEKKMFRIRNRSYIDRDSEYLLQENEPDGTLDQKLLEDLQKKK
NDLRYIEMQHFREKLPSYGMQKELVNLIDNHQVTVISGETGCGKTTQVTQFILDNYIERGKGSACRI
VCTQPRRISAISVAERVAAERAESCGSGNSTGYQIRLQSRLPRKQGSILYCTTGIILQWLQSDPYLSSVS
HIVLDEIHERNLQSDVLMTVVKDLLNFRSDLKVILMSATLNAEKFSEYFGNCPMIHIPGFTFPVVEYL
LEDVIEKIRYVPEQKEHRSQFKRGFMQGHVNRQEKEEKEAIYKERWPDYVRELRRRYSASTVDVIEM
MEDDKVDLNLIVALIRYIVLEEEDGAILVFLPGWDNISTLHDLLMSQVMFKSDKFLIIPLHSLMPTVN
QTQVFKRTPPGVRKIVIATNIAETSITIDDVVYVIDGGKIKETHFDTQNNISTMSAEWVSKANAKQR
KGRAGRVQPGHCYHLYNGLRASLLDDYQLPEILRTPLEELCLQIKILRLGGIAYFLSRLMDPPSNEAV
LLSIRHLMELNALDKQEELTPLGVHLARLPVEPHIGKMILFGALFCCLDPVLTIAASLSFKDPFVIPLG
KEKIADARRKELAKDTRSDHLTVVNAFEGWEEARRRGFRYEKDYCWEYFLSSNTLQMLHNMKGQ
FAEHLLGAGFVSSRNPKDPESNINSDNEKIIKAVICAGLYPKVAKIRLNLGKKRKMVKVYTKTDGLV
AVHPKSVNVEQTDFHYNWLIYHLKMRTSSIYLYDCTEVSPYCLLFFGGDISIQKDNDQETIAVDEWI
VFQSPARIAHLVKELRKELDILLQEKIESPHPVDWNDTKSRDCAVLSAIIDLIKTQEKATPRNFPPRFQ

DGYYS

**>DHX36 isoform 2**

MSYDYHQNWGRDGGPRSSGGGYGGGPAGGHGGNRGSGGGGGGGGGRGGRGRHPGHLKGREI
GMWYAKKQGQKNKEAERQERAVVHMDERREEQIVQLLNSVQAKNDKESEAQISWFAPEDHGYG
TEVSTKNTPCSENKLDIQEKKLINQEKKMFRIRNRSYIDRDSEYLLQENEPDGTLDQKLLEDLQKKK
NDLRYIEMQHFREKLPSYGMQKELVNLIDNHQVTVISGETGCGKTTQVTQFILDNYIERGKGSACRI
VCTQPRRISAISVAERVAAERAESCGSGNSTGYQIRLQSRLPRKQGSILYCTTGIILQWLQSDPYLSSVS
HIVLDEIHERNLQSDVLMTVVKDLLNFRSDLKVILMSATLNAEKFSEYFGNCPMIHIPGFTFPVVEYL
LEDVIEKIRYVPEQKEHRSQFKRGFMQGHVNRQEKEEKEAIYKERWPDYVRELRRRYSASTVDVIEM
MEDDKVDLNLIVALIRYIVLEEEDGAILVFLPGWDNISTLHDLLMSQVMFKSVNQTQVFKRTPPGV
RKIVIATNIAETSITIDDVVYVIDGGKIKETHFDTQNNISTMSAEWVSKANAKQRKGRAGRVQPGHC
YHLYNGLRASLLDDYQLPEILRTPLEELCLQIKILRLGGIAYFLSRLMDPPSNEAVLLSIRHLMELNAL
DKQEELTPLGVHLARLPVEPHIGKMILFGALFCCLDPVLTIAASLSFKDPFVIPLGKEKIADARRKEL
AKDTRSDHLTVVNAFEGWEEARRRGFRYEKDYCWEYFLSSNTLQMLHNMKGQFAEHLLGAGFVS
SRNPKDPESNINSDNEKIIKAVICAGLYPKVAKIRLNLGKKRKMVKVYTKTDGLVAVHPKSVNVEQ
TDFHYNWLIYHLKMRTSSIYLYDCTEVSPYCLLFFGGDISIQKDNDQETIAVDEWIVFQSPARIAHLV
KELRKELDILLQEKIESPHPVDWNDTKSRDCAVLSAIIDLIKTQEKATPRNFPPRFQDGYYS

**>SRSF1 isoform1**

MSGGGVIRGPAGNNDCRIYVGNLPPDIRTKDIEDVFYKYGAIRDIDLKNRRGGPPFAFVEFEDPRDA
EDAVYGRDGYDYDGYRLRVEFPRSGRGTGRGGGGGGGGGAPRGRYGPPSRRSENRVVVSGLPPSG
SWQDLKDHMREAGDVCYADVYRDGTGVVEFVRKEDMTYAVRKLDNTKFRSHEGETAYIRVKVD
GPRSPSYGRSRSRSRSRSRSRSRSNSRSRSYSPRRSRGSPRYSPRHSRSRSRT

**>SRSF9**

MSGWADERGGEGDGRIYVGNLPTDVREKDLEDLFYKYGRIREIELKNRHGLVPFAFVRFEDPRDAE
DAIYGRNGYDYGQCRLRVEFPRTYGGRGGWPRGGRNGPPTRRSDFRVLVSGLPPSGSWQDLKDH
MREAGDVCYADVQKDGVGMVEYLRKEDMEYALRKLDDTKFRSHEGETSYIRVYPERSTSYGYSRSR
SGSRGRDSPYQSRGSPHYFSPFRPY

**>TLS**

MASNDYTQQATQSYGAYPTQPGQGYSQQSSQPYGQQSYSGYSQSTDTSGYGQSSYSSYGQSQNTGY
GTQSTPQGYGSTGGYGSSQSSQSSYGQQSSYPGYGQQPAPSSTSGSYGSSSQSSSYGQPQSGSYSQQPS
YGGQQQSYGQQQSYNPPQGYGQQNQYNSSSGGGGGGGGGGNYGQDQSSMSSGGGSGGGYGNQ
DQSGGGGSGGYGQQDRGGRGRGGSGGGGGGGGGGYNRSSGGYEPRGRGGGRGGRGGMGGSDR
GGFNKFGGPRDQGSRHDSEQDNSDNNTIFVQGLGENVTIESVADYFKQIGIIKTNKKTGQPMINLY
TDRETGKLKGEATVSFDDPPSAKAAIDWFDGKEFSGNPIKVSFATRRADFNRGGGNGRGGRGRGG
PMGRGGYGGGGSGGGGRGGFPSGGGGGGGQQRAGDWKCPNPTCENMNFSWRNECNQCKAPK
PDGPGGGPGGSHMGGNYGDDRRGGRGGYDRGGYRGRGGDRGGFRGGRGGGDRGGFGPGKMDS
RGEHRQDRRERPY

**>Telomeric repeat-binding factor 2**

MAAGAGTAGPASGPGVVRDPAASQPRKRPGREGGEGARRSDTMAGGGGSSDGSGRAAGRRASRS
SGRARRGRHEPGLGGPAERGAGEARLEEAVNRWVLKFYFHEALRAFRGSRYGDFRQIRDIMQALL
VRPLGKEHTVSRLLRVMQCLSRIEEGENLDCSFDMEAELTPLESAINVLEMIKTEFTLTEAVVESSRK
LVKEAAVIICIKNKEFEKASKILKKHMSKDPTTQKLRNDLLNIIREKNLAHPVIQNFSYETFQQKMLR
FLESHLDDAEPYLLTMAKKALKSESAASSTGKEDKQPAPGPVEKPPREPARQLRNPPTTIGMMTLK
AAFKTLSGAQDSEAAFAKLDQKDLVLPTQALPASPALKNKRPRKDENESSAPADGEGGSELQPKN

KRMTISRLVLEEDSQSTEPSAGLNSSQEAASAPPSKPTVLNQPLPGEKNPKVPKGKWNSSNGVEEKE
TWVEEDELFQVQAAPDEDSTTNITKKQKWTVEESEWVKAGVQKYGEGNWAAISKNYPFVNRTAV
MIKDRWRTMKRLGMN
